# Disease trajectory and competing risks of patients with cirrhosis in the US

**DOI:** 10.1371/journal.pone.0313152

**Published:** 2025-02-14

**Authors:** Mohsen Mohammadi, Bima J. Hasjim, Salva N. Balbale, Praneet Polineni, Alexander A. Huang, Mitchell Paukner, Therese Banea, Oriana Dentici, Dominic J. Vitello, Joy E. Obayemi, Andrés Duarte-Rojo, Satish N. Nadig, Lisa B. VanWagner, Lihui Zhao, Sanjay Mehrotra, Daniela P. Ladner

**Affiliations:** 1 Northwestern University Transplant Outcomes Research Collaborative (NUTORC), Comprehensive Transplant Center (CTC), Northwestern University, Chicago, IL, United States of America; 2 Department of Industrial Engineering and Management Sciences, McCormick School of Engineering, Northwestern University, Evanston, IL, United States of America; 3 Division of Gastroenterology and Hepatology, Department of Medicine, Northwestern University, Chicago, IL, United States of America; 4 Department of Surgery, Center for Health Services and Outcomes Research, Institute of Public Health and Medicine & Northwestern Quality Improvement, Research, & Education in Surgery (NQUIRES), Northwestern University Feinberg School of Medicine, Chicago, IL, United States of America; 5 Center of Innovation for Complex Chronic Healthcare (CINCCH), Edward Hines, Jr. VA Hospital, Hines, IL, United States of America; 6 Division of Biostatistics, Department of Preventative Medicine, Northwestern University, Chicago, IL, United States of America; 7 Division of Organ Transplantation, Department of Surgery, Northwestern University, Chicago, IL, United States of America; 8 Division of Digestive and Liver Diseases, Department of Medicine, University of Texas Southwestern Medical Center, Dallas, TX, United States of America; Al-Azhar University, EGYPT

## Abstract

**Background:**

Cirrhosis is a dynamic disease process leading to liver-related death, which has increased by over 65% over the last decade. Unpredictable hepatic decompensation complications are a major source of morbidity and mortality. Thus, accurately characterizing disease progression through discrete stages of cirrhosis is critical towards implementing timely intervention and liver transplant (LT) waitlisting.

**Methods:**

A retrospective, longitudinal, population-cohort study of adult patients with cirrhosis from a US metropolitan area (2006–2012) was conducted. Clinical diagnoses were defined by ICD-9 and CPT codes. Cirrhosis stages were defined as: compensated without portal hypertension (Stage 1), compensated with portal hypertension (Stage 2), variceal bleeding (Stage 3), hepatic encephalopathy (Stage 4a), ascites (Stage 4b), and ≥2 different decompensating complications (Stage 5). Multivariate Fine-Gray competing risk survival analysis adjusted for clinicodemographic covariates.

**Results:**

Among 12,196 patients with cirrhosis, the mean (±SD) age was 56.8 (±11.7) years with a follow-up time of 2.35 (±1.81) years. A novel 5-stage disease progression framework was used. The 1-year mortality rates for each stage were 7.3% for Stage 1, 5.4% for Stage 2, 11.4% for Stage 3, 10.0% for Stage 4a, 20.2% for Stage 4b, and 43.8% for Stage 5. Compared to those in Stage 1, Stage 3 (sHR:1.83, 95% CI:1.36–2.48, P<0.001), Stage 4b (sHR:1.45, 95% CI:1.23–1.70, P<0.001), and Stage 5 (sHR:1.95, 95% CI:1.71–2.23, P<0.001) patients had higher risks of mortality. Additional disease progression rates were identified.

**Conclusion:**

Even among patients with compensated cirrhosis, the 1-year mortality rate was as high as 7.3% and subsequently increases with each decompensation complication. This one-year mortality rate is higher than 5-years mortality rate reported in previously known non-US studies. The highest associated risk of death was observed among patients with ≥2 different decompensating complications (95.2%), variceal bleeding (83.2%) and ascites (44.9%). Overall, patients in advanced stages of cirrhosis were more likely to die than they were to receive a LT, suggesting that patients should be referred and waitlisted for LT earlier in the disease process.

## Introduction

Cirrhosis is a major public health burden that affects approximately 2–8 million adults in the United States (US) [1–3]. Hepatic decompensation complications (e.g., ascites, variceal bleeding, hepatic encephalopathy [HE]) arise in 5–7% of patients with compensated cirrhosis within a year of diagnosis and precipitously decreases the median survival from 12 years to 1.8 years [4, 5]. Cirrhosis progression is dynamic, and each associated complication has unique risks of morbidity and mortality. For example, variceal bleeding is associated with a 21% increased risk of mortality while spontaneous bacterial peritonitis (SBP) harbors a 4-fold increase [4, 6, 7].

The unique mortality risks of each decompensating complication led to the Baveno VII consensus 5-stage model for cirrhosis based on D’Amico et al.’s, longitudinal cohort study of 494 patients from Italy. These stages include: compensated without portal hypertension (Stage 1), compensated with portal hypertension (Stage 2), variceal bleeding (Stage 3), non-bleeding decompensation complication (Stage 4), and ≥2 decompensation complications (Stage 5) [7, 8]. The staging framework captures the cumulative disease burden of cirrhosis, which is unique from clinical scores, like the Model for End-stage Liver Disease with Sodium (MELD-Na) or Child-Turcotte-Pugh (CTP), that characterize a patient’s clinical status from only a single snapshot in time [9, 10]. Additionally, elucidating the trajectory of disease through discrete stages may help guide timing of preventative medical interventions and therapeutic treatments (e.g., liver transplantation [LT]) [11]. However, current studies describing the discrete 5-stage model of cirrhosis are limited to small sample size, outdated, or highly select (e.g., LT waitlisted patients) cohorts [6, 7, 9]. Thus, these studies may not be representative of current disease distribution and standard of care in the US.

We used a longitudinal, diverse, and more contemporary cohort of patients with cirrhosis from a large US metropolitan area to examine the distribution of disease stages, LT, and mortality. Accurately characterizing the natural history of cirrhosis is vital to prognosticating the progression of disease and anticipating timely interventions.

## Materials and methods

### Study design

This was a retrospective, longitudinal, population-cohort study using the HealthLNK data repository of electronic health records (EHR) from January 1^st^, 2006 through December 31^st^, 2012. The Strengthening the Reporting of Observational Studies in Epidemiology (STROBE) guideline was used to describe the natural history of patients with cirrhosis [12]. This study was approved by the Northwestern University Institutional Review Board (IRB ID # STU00213447) and also waived the need for patient informed consent due to its retrospective nature.

### Data source

The HealthLNK database is a de-duplicated database from six health care institutions in the greater metropolitan Chicago area and includes: Northwestern Medicine, University of Chicago Hospitals and Clinic, Rush University Medical Center, University of Illinois at Chicago Medical Center, Loyola University Medical Center, and Cook County Health and Hospitals System. The United Network for Organ Sharing (UNOS) database is a national registry of all patients waitlisted for organ transplantation in the US. Information on mortality was extracted from the Social Security Death Master File for the state of Illinois. Both databases were merged with the HealthLNK database in order to extract LT, waitlist, and mortality data as previously described [13, 14].

### Setting and subjects

**Fig 1:**
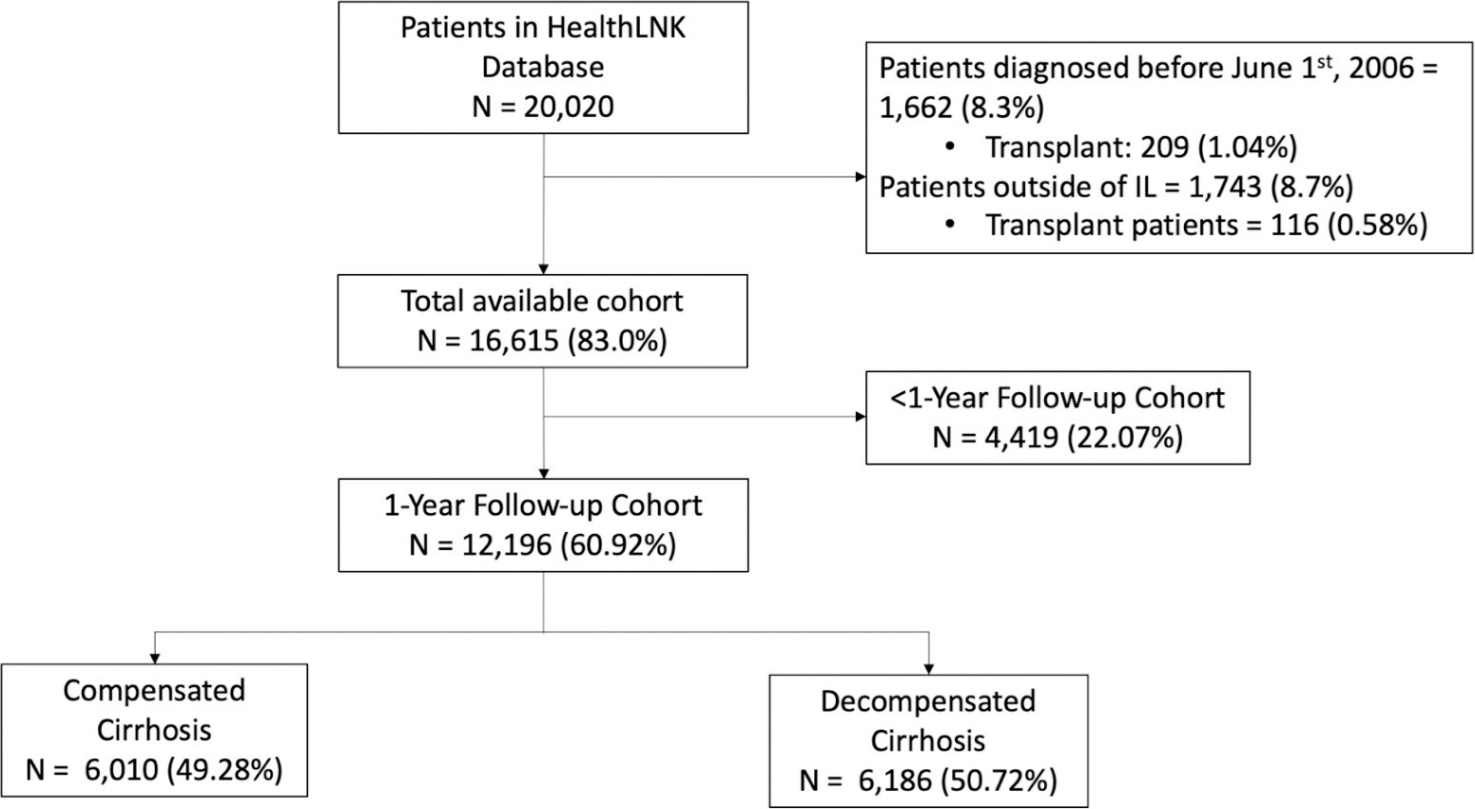
Flowchart for cohort selection from the HealthLNK database.

Adult patients (≥18-years-old) with cirrhosis were included in the study. Cirrhosis was defined as having one of the International Classification of Diseases, 9th Revision (ICD-9) and Current Procedural Terminology (CPT) codes as previously published (*S1 Table*) [15, 16]. Patients were excluded from the study if they were under 18 years old, resided outside of Illinois, or had a cirrhosis inclusion code that preceded a liver transplant (LT) (Fig 1). Patients who develop cirrhosis after a LT are different from those who have cirrhosis the first time and were therefore excluded. The first appearance of a cirrhosis code was considered the index date. Only patients with an index date after June 1^st^, 2006 (6 months after database inception) were included to ensure that the index date was the first time a patient was diagnosed with cirrhosis. The follow-up period for patients is defined as the duration from their first cirrhosis diagnosis until their last record (ICD, CPT, medications, labs) in the HealthLNK dataset. In addition, the follow-up period concludes if the patient receives a censoring event (LT or death). Patients without 1 year of follow-up in the HealthLNK healthcare network (any ICD codes, medications, procedure codes) were removed from 1-year outcome analyses. The time between healthcare touchpoints refers to the interval between consecutive healthcare interactions recorded in the database for a patient. This measure serves as a surrogate for how frequently patients were monitored within our dataset. Understanding these intervals helps to assess the frequency of patient care and the potential impact on clinical outcomes.

### Covariates

Demographic information was captured at the index date and included age, sex, race/ethnicity, and insurance type. Etiologies of cirrhosis were defined by ICD-9 and included alcohol-associated, metabolic dysfunction associated steatohepatitis (MASH, formally termed non-alcoholic steatohepatitis [NASH]), hepatitis C virus (HCV), cholestasis (e.g., primary sclerosing cholangitis, primary biliary cirrhosis), hepatitis B virus (HBV), autoimmune, hemochromatosis, and Wilson’s disease. MASH was defined as those coded for non-alcoholic associated cirrhosis who did not have any code for alcohol-associated, HCV, HBV, biliary cirrhosis, autoimmune hepatitis, Wilson’s disease, or hemochromatosis, but also had diagnosis codes for obesity and diabetes, or hypertension [17].

Decompensating complications were defined as the occurrence of any of the following complications identified by ICD-9, CPT codes, or medications at any point during the observation period: HE, ascites, SBP, variceal bleeding, hepatorenal syndrome (HRS), or hepatopulmonary syndrome (HPS) (*S1 Table*) [18]. Patients were classified as compensated if they did not meet any of the prescribed medications or EHR codes for decompensation during the entirety of their follow-up from index cirrhosis diagnosis.

The MELD-Na, the Charlson Comorbidity Index, and each comorbidity were defined as previously published [15, 16, 19]. The MELD-Na score was calculated with the four components of lab data (serum creatinine, bilirubin, international normalized ratio [INR], and sodium) when they were present within 60 days of each other and the cirrhosis inclusion code. Fibrosis-4 (FIB-4) scores were calculated with platelets, AST, ALT, and albumin measured within 60 days of each other [20]. Missing laboratory results were encountered in 10,223 (61.5%) subjects for MELD-Na, and 7,712 (46.4%) subjects for FIB-4.

### Study outcomes

The primary aim of our study was to describe the rates and risk of mortality of each cirrhosis stage. Secondary outcomes included assessing the rates of transition from each stage and LT. Manual death certificate review of the Social Security Death Master File of Illinois was performed by a panel of clinicians in which they were blinded to all other patient information. Causes of death were categorized into liver-related, non-liver related, and non-descript. Non-descript causes of death (e.g., cardiac arrest) were defined as those missing a cause of death or without identifiable associations of disease. If the decision was not unanimous, the death certificate was flagged for further review and was eventually classified based on ordinal review of contributing causes indicated in each death certificate.

### Cirrhosis Progression Stages

**Fig 2:**
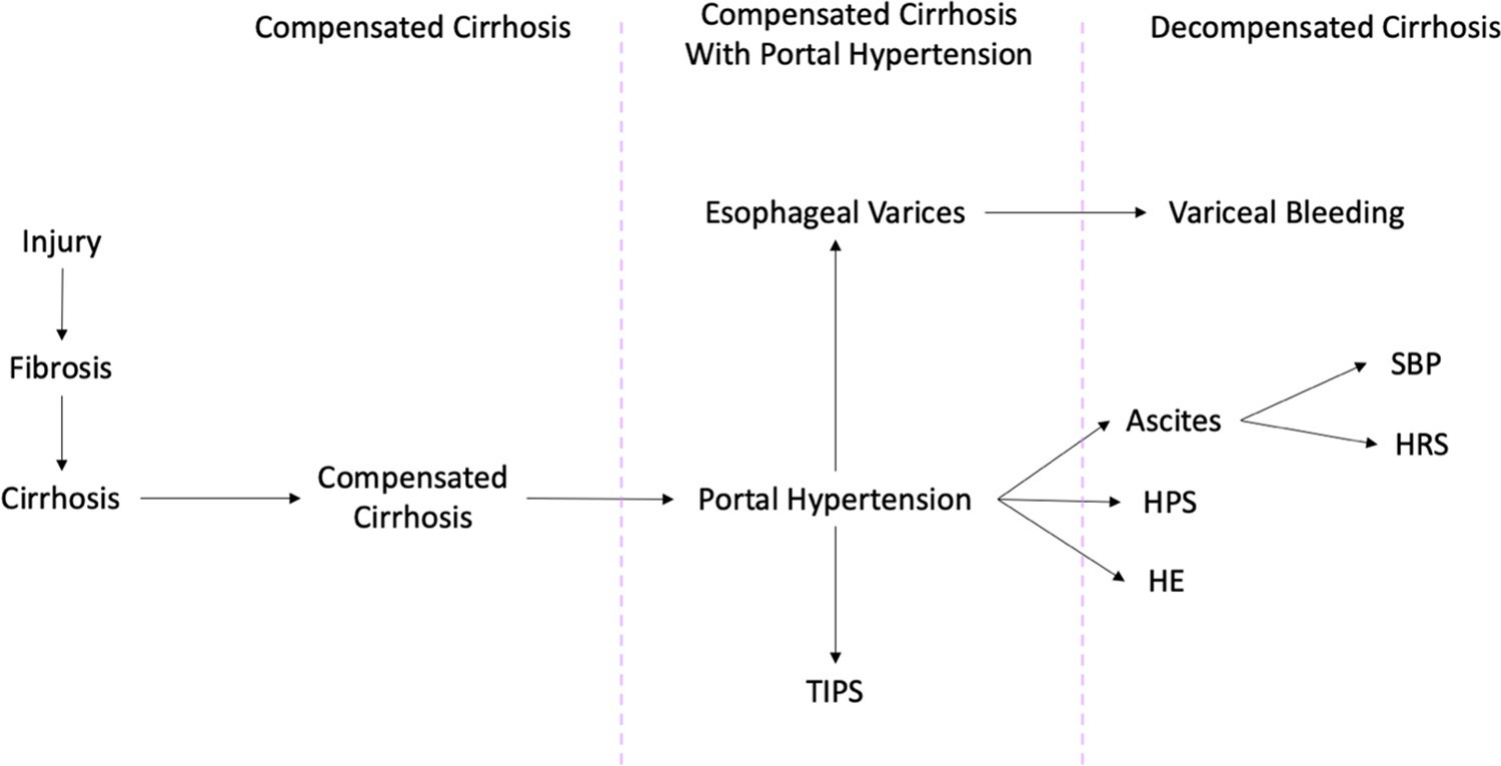
Diagram of the pathophysiological representation of cirrhosis trajectory. HE: hepatic Encephalopathy, HPS: hepatopulmonary Syndrome, HRS: hepatorenal syndrome, SBP: spontaneous bacterial peritonitis, TIPS: transjugular intrahepatic portosystemic shunt.

Cirrhosis stages were categorized into 5 stages as outlined by D’Amico et al. (2014) and the Baveno VII consensus with modifications based on the pathophysiology of cirrhosis (*Fig 2*) [7, 8, 21].

- Stage 1: Compensated cirrhosis without portal hypertension
- Stage 2: Compensated cirrhosis with portal hypertension (esophageal varices, or platelets <100 x 10^9^/L)
- Stage 3: Variceal bleeding
- Stage 4: HE (Stage 4a), ascites (Stage 4b), HPS (Stage 4c)
- Stage 5: SBP, HRS, or ≥2 different decompensating complication

The Cirrhosis Progression Stages represents the patient’s EHR codes as a diagram to illustrate the patient’s disease progression within 1 year. The 1-year follow-up endpoint was used to mitigate the differences in times of follow-up among patients. The highest resolution of time in the HealthLNK database was at the month level, thus clinical events that occur within the same month were assumed to occur concurrently. Nodes of the graph may be diagnostic or therapeutic events and follow a chain of connected nodes representing each stage. Patients may start at any stage depending on the associated clinical diagnoses that were coded at index of cirrhosis diagnosis. For example, a patient who starts in Stage 1 is a patient who has a cirrhosis inclusion code without any other cirrhosis-related decompensation or portal hypertension codes. SBP and HRS were considered to be Stage 5 complications since they cannot occur without ascites [21]. If a patient received a LT in the same month as a decompensating complication, it was assumed that the decompensating complication occurred prior to LT. Terminal stages of the Cirrhosis Progression Stages were LT and death.

### Statistical analyses

Demographic variables were compared using Kruskal-Wallis and Pearson’s chi-squared test for continuous and categorical variables, respectively. Means were reported with standard deviations (±SD) while medians were reported with its interquartile range (IQR). For all results, two-sided P-values were used and those with a p-value of <0.05 were considered statistically significant.

Fine-Gray multivariate, competing risk survival analysis was conducted with all-cause mortality and liver-related death as the events of interest and LT as its competing event. Competing risk models adjusted for known predictors of mortality in patients with cirrhosis: age, race, sex, cirrhosis etiology, initial stage of disease, hepatocellular carcinoma (HCC), Charlson Comorbidity Index, and the median laboratory results (ALT, AST, platelet, bilirubin, albumin, INR, sodium, creatinine) over the follow-up period. Patients were included in the competing risk analysis only if they had all the available covariates needed for analysis and no imputations were used in the analysis to minimize bias. Patients with HPS were not included in the Cirrhosis Progression Stage graph, nor competing risk analysis as there was a very small sample size (N = 10, 0.06%) and had missing covariates. Results were expressed in subdistribution hazard ratios (sHR) with 95% confidence intervals (95% CI). Cumulative incidence graphs stratified by the cirrhosis stages were constructed to graphically represent the competing risk model. Data processing and analysis were performed using R studio (version 4.1.0).

## Results

### Cohort characteristics

Among 16,615 patients with cirrhosis, there were 12,196 (60.9%) patients with at least 1 year of follow-up. The mean (±SD) age was 56.8 (±11.7) years, follow-up time was 28.15 (±21.74) months, and time between healthcare touchpoints was 61.8 (±30.8) days. Of these, 5,166 (42.4%) patients were women, 5,767 (47.3%) were Non-Hispanic White, 2,975 (24.4%) were Non-Hispanic Black, 710 (5.8%) were Hispanic, and 347 (2.9%) were Asian. There were 6,557 (53.8%) patients enrolled in Medicare/Medicaid, 3,252 (26.7%) enrolled in private insurance, and 2,388 (19.6%) enrolled in Other insurance. The most frequent etiologies of cirrhosis were HCV (N = 4,859, 39.8%), alcohol-associated (N = 3,929, 32.2%), and MASH (N = 2,408, 19.7%). The mean Charlson Comorbidity Index was 5.9 (3.2), median (IQR) MELD-Na was 13 (9–20), and FIB-4 score was 4.4 (2.3–7.7). Within 1 year of follow-up, 2,701 (22.2%) of patients died: 1,745 (14.3%) from liver-related deaths, 671 (5.5%) from non-liver related deaths, and 285 (2.3%) from non-descript deaths. Demographic statistics were stratified by initial cirrhosis stage and there was a higher incidence of mortality with increasing stage (P<0.001) (*Table 1*).

**Table 1:** Demographics and 1-year outcomes of patients with cirrhosis stratified by initial stage.

	All (n = 12,196)	Compensated Cirrhosis		Decompensated Cirrhosis			p-value
Characteristic		Stage I	Stage II	Stage III	Stage IV	Stage V	
		(n = 6,070)	(n = 1,840)	(n = 387)	(n = 2,032)	(n = 1,867)	
Age, year, mean (±SD)	56.79(11.68)	57.17 (11.99)	55.17 (10.72)	56.61 (12.07)	57.61 (11.88)	56.33 (11.08)	<0.001
Female, n (%)	5166(42.36%)	2831 (46.64%)	692 (37.61%)	145 (37.47%)	838 (41.24%)	660 (35.35%)	<0.001
Race, n (%)							
Non-Hispanic White	5767(47.29%)	2887 (47.56%)	772 (41.96%)	141 (36.43%)	990 (48.72%)	977 (52.33%)	<0.001
Black	2975(24.39%)	1539 (25.35%)	394 (21.41%)	125 (32.3%)	560 (27.56%)	357 (19.12%)	<0.001
Hispanic	710(5.82%)	294 (4.84%)	179 (9.73%)	41 (10.59%)	101 (4.97%)	95 (5.09%)	0.009
Asian	347(2.85%)	166 (2.73%)	74 (4.02%)	12 (3.1%)	42 (2.07%)	53 (2.84%)	<0.001
Other	2398(19.66%)	1184 (19.51%)	422 (22.93%)	68 (17.57%)	339 (16.68%)	385 (20.62%)	<0.001
Insurance, n (%)							
Medicare/Medicaid	6557(53.76%)	3283 (54.09%)	896 (48.7%)	203 (52.45%)	1168 (57.48%)	1007 (53.94%)	<0.001
Private	3252(26.66%)	1894 (31.2%)	391 (21.25%)	73 (18.86%)	433 (21.31%)	461 (24.69%)	<0.001
Other	2388(19.58%)	893 (14.71%)	554 (30.05%)	111 (28.69%)	431 (21.21%)	399 (21.37%)	<0.001
Follow-up time (months), mean (±SD)	28.15(21.74)	31.58(21.11)	32.13(20.31)	30.07(22.16)	24.59(21.89)	16.56(20.17)	<0.001
Time between healthcare touch points (days), mean (±SD)	61.81(30.83)	70.52(34.96)	60.99(32.46)	57.09(28.95)	50.85(23.29)	44.86(21.71)	<0.001
Charlson Comorbidity Index, mean (±SD)	5.91(3.22)	4.67 (3.1)	7.35 (2.48)	6.4 (2.95)	6.62 (3.3)	7.68 (2.47)	<0.001
Etiology, n (%)							
Hepatitis C	914(7.49%)	2476 (40.79%)	846 (45.98%)	142 (36.69%)	770 (37.89%)	625 (33.48%)	0.072
Alcohol-related	4859(39.84%)	1361 (22.42%)	584 (31.74%)	157 (40.57%)	738 (36.32%)	1089 (58.33%)	<0.001
MASH	3929(32.22%)	1196 (19.7%)	316 (17.17%)	84 (21.71%)	498 (24.51%)	309 (16.55%)	<0.001
Hepatitis B	2408(19.74%)	474 (7.81%)	140 (7.61%)	24 (6.2%)	142 (6.99%)	134 (7.18%)	<0.001
Cholestasis	804(6.59%)	635 (10.46%)	47 (2.55%)	13 (3.36%)	57 (2.81%)	52 (2.79%)	<0.001
Autoimmune	282(2.31%)	170 (2.8%)	43 (2.34%)	10 (2.58%)	31 (1.53%)	28 (1.5%)	0.001
Genetic	125(1.02%)	68 (1.12%)	14 (0.76%)	2 (0.52%)	21 (1.03%)	20 (1.07%)	0.490
Labs							
MELD-Na, median (IQR)	13([9,20])	10 (7,15)	12 (9,16)	12 (8,17)	16 (11,21)	22 (16,28)	<0.001
Fib-4, median (IQR)	4.36([2.27,7.73])	2.59 (1.52,4.18)	7.32 (4.94,11.13)	4.48 (2.71,7.37)	2.99 (1.77,4.91)	7.04 (4.01,11.34)	<0.001
Platelets, median (IQR)	130([85,204])	181 (134,245)	79 (62,93)	136 (85,191)	182 (137,251)	100 (69,164)	<0.001
AST, median (IQR)	57([35,100])	48 (31,82)	69 (44,109)	58 (35,98.5)	53 (32,92)	71 (45,126)	<0.001
ALT, median (IQR)	38([24,67])	39 (24,70)	45 (29,78)	33 (22.5,60)	32 (20,57)	36 (23,61)	<0.001
Albumin, median (IQR)	3.2([2.6,3.7])	3.5 (3,3.9)	3.3 (2.8,3.7)	3.1 (2.6,3.7)	3 (2.4,3.5)	2.5 (2.1,3)	<0.001
Decompensation, n (%)							<0.001
Ascites	4709(38.61%)	901 (14.84%)	461 (25.05%)	84 (21.71%)	1592 (78.35%)	1671 (89.5%)	<0.001
SBP	683(5.6%)	94 (1.55%)	64 (3.48%)	9 (2.33%)	116 (5.71%)	400 (21.42%)	<0.001
HE	3155(25.87%)	563 (9.28%)	310 (16.85%)	62 (16.02%)	875 (43.06%)	1345 (72.04%)	<0.001
Variceal Bleeding	1676(13.74%)	287 (4.73%)	211 (11.47%)	387 (100%)	199 (9.79%)	592 (31.71%)	<0.001
HRS	612(5.02%)	81 (1.33%)	44 (2.39%)	9 (2.33%)	87 (4.28%)	391 (20.94%)	<0.001
HPS	10(0.08%)	1 (0.02%)	2 (0.11%)	0 (0%)	2 (0.1%)	5 (0.27%)	0.801
HCC, n (%)	1780(14.59%)	786 (12.95%)	364 (19.78%)	43 (11.11%)	293 (14.42%)	294 (15.75%)	<0.001
Transplant, n (%)	229(1.88%)	79 (1.3%)	51 (2.77%)	1 (0.26%)	24 (1.18%)	74 (3.96%)	<0.001
Death, n (%)		760 (12.5%)	226 (12.3%)	87 (22.5%)	666 (32.8%)	962 (51.5%)	
Liver-related	1745(14.31%)	432 (7.12%)	155 (8.42%)	45 (11.63%)	383 (18.85%)	730 (39.1%)	<0.001
Non-Liver	671(5.5%)	243 (4%)	60 (3.26%)	26 (6.72%)	189 (9.3%)	153 (8.19%)	<0.001
Non-descript	285(2.34%)	85 (1.4%)	11 (0.6%)	16 (4.13%)	94 (4.63%)	79 (4.23%)	<0.001

ALT: Alanine transaminase, AST: aspartate transaminase, FIB-4 = fibrosis-4 score, HCC: hepatocellular carcinoma, HPS: hepatopulmonary Syndrome, HRS: hepatorenal syndrome, MASH: metabolic dysfunction associated steatohepatitis, SBP: spontaneous bacterial peritonitis, Stage 1: compensated cirrhosis without portal hypertension, Stage 2: compensated cirrhosis with portal hypertension, Stage 3: variceal bleeding, Stage 4: non-bleeding decompensation complication, Stage 5: ≥2 different decompensation complications.

### 1-year Cirrhosis Progression Stages

**Fig 3:**
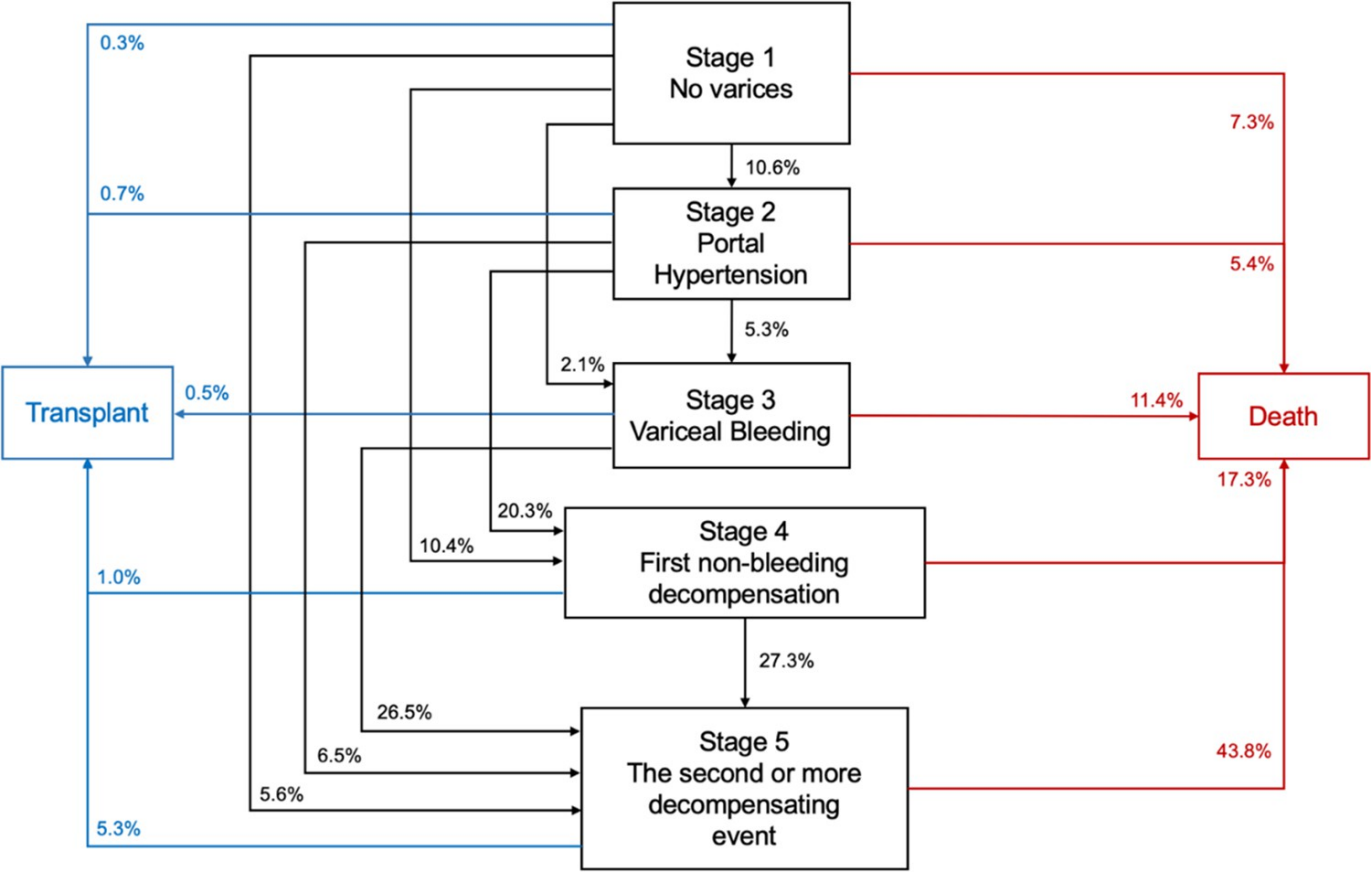
1-year Cirrhosis Progression Stages diagram. Progression of cirrhosis over 1-year follow-up from index cirrhosis diagnosis based on the Baveno VII consensus stages of cirrhosis. Proportion of patients that advance from each stage towards higher stage, transplant, or death.

The Cirrhosis Progression Stages details the progression of cirrhosis within 1-year follow-up (*Fig 3*). Of patients in Stage 1, 28.6% advanced to a higher stage (10.6% to Stage 2; 2.1% to Stage 3; 10.4% to Stage 4; 5.6% to Stage 5), 0.3% received LT, and 7.3% died. Of patients in Stage 2, 32.0% advanced to a higher stage (5.3% to Stage 3; 20.3% to Stage 4; 5.6% to Stage 5), 0.7% received LT, and 5.4% died. Cumulatively among patients with compensated cirrhosis (Stage 1 and Stage 2), the 1-year LT and mortality was 0.4% and 6.7%, respectively (*Fig 3*).

The 1-year rates of transition for Stage 3 patients who advanced to Stage 5, received LT, and died were 26.5% (0.5%, and 11.4%, respectively). Of patients in Stage 4, 27.3% advanced to Stage 5 (24.6% from Stage 4a; 28.3% from Stage 4b), 1.0% received LT (0.4% from Stage 4a; 1.2% from Stage 4b), and 17.3% died (10.0% from Stage 4a; 20.2% from Stage 4b). There were 16.3% of patients with 1 decompensating event who died within 1 year (11.4% from Stage 3; 17.3% from Stage 4). The 1-year mortality for patients in Stage 5 was 43.8%, while 5.3% received LT (*Fig 3*).

### Competing risk analysis: Mortality and liver-related death

**Fig 4:**
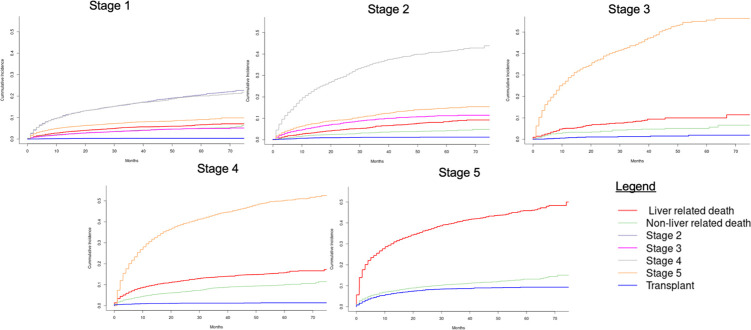
Cumulative incidence graph of competing risk analysis for mortality, stage progression and/or transplant. Cumulative incidence graphs of competing risk analysis with liver transplantation, non-liver related death, and liver-related death as the competing risks of interest. Patients in Stages 1–4 also had the proceeding higher stages as additional competing risks events. At all stages, even in patients with decompensating complications, patients had higher rates of mortality than LT. LT: liver transplant, Stage 1: without portal hypertension, Stage 2: with portal hypertension, Stage 3: bleeding, Stage 4: non-bleeding decompensation complication, Stage 5: ≥2 different decompensation complications.

Cumulative incidence curves in *Fig 4* illustrate the rates of each competing event. At all stages of disease, even in those with decompensating events, patients were more likely to die than they were to receive LT (*Fig 4*).

All-cause mortality: Compared to Stage 1 cirrhosis, patients with Stage 3 (sHR:1.83, 95% CI:1.35–2.48, P<0.001), Stage 4b (sHR:1.45, 95% CI:1.23–1.70, P<0.001), and Stage 5 (sHR:1.95, 95% CI:1.71–2.23, P<0.001) disease have increased subdistribution hazards for all-cause mortality. There were no differences in the subdistribution hazards of all-cause mortality for Stage 2 (sHR:1.06, 95% CI:0.91–1.23, P = 0.460) or Stage 4a (sHR:1.23, 95% CI:0.95–1.61, P = 0.120) compared to Stage 1. HCC was associated with an increased subdistribution hazard of all-cause mortality (sHR:1.51, 95% CI:1.33–1.71, P<0.001) (*Table 2*).

**Table 2:** Competing risk analysis of patients with cirrhosis identifying predictors of all-cause mortality and liver-related death.

	All-cause mortality		Liver-related death	
	sHR	95% CI	sHR	95% CI
Initial stage	** **	** **	** **	** **
Stage 1	Reference	** **	Reference	** **
Stage 2	1.06	[0.91,1.23]	1.05	[0.88,1.25]
Stage 3	1.83***	[1.35,2.48]	1.66**	[1.16,2.38]
Stage 4a	1.23	[0.95,1.61]	1.15	[0.83,1.59]
Stage 4b	1.45***	[1.23,1.70]	1.24*	[1.02,1.51]
Stage 5	1.95***	[1.71,2.23]	1.85***	[1.58,2.18]
Age	1.02***	[1.02,1.03]	1.02***	[1.01,1.03]
Male (female is reference)	1.06	[0.95,1.18]	1.00	[0.88,1.14]
Charlson	1.07***	[1.05,1.09]	0.99	[0.97,1.01]
HCC (absence is reference)	1.51***	[1.33,1.71]	2.85***	[2.49,3.27]
ALT	1.00	[0.99,1.00]	1.00	[0.99,1.00]
AST	1.00***	[1.00,1.00]	1.00**	[1.00,1.00]
Platelet	1.00***	[1.00,1.00]	1.00	[1.00,1.00]
Bilirubin	1.04***	[1.03,1.04]	1.04***	[1.03,1.05]
Albumin	0.40***	[0.36,0.43]	0.40***	[0.36,0.44]
INR	1.37***	[1.23,1.54]	1.35***	[1.18,1.53]
Serum sodium	0.95***	[0.93,0.97]	0.93***	[0.91,0.95]
Serum creatinine	1.25***	[1.18,1.32]	1.24***	[1.16,1.33]

ALT: Alanine transaminase, AST: aspartate transaminase, HCC: hepatocellular carcinoma, INR: international normalized ratio, Stage 1: compensated cirrhosis without portal hypertension, Stage 2: compensated cirrhosis with portal hypertension, Stage 3: variceal bleeding, Stage 4a: hepatic encephalopathy, Stage 4b: ascites, Stage 5: ≥2 different decompensation complications

Adjusted for known predictors of mortality in patients with cirrhosis such as age, race, sex, cirrhosis etiology, initial cirrhosis stage, HCC, Charlson Comorbidity Index and median laboratory results (ALT, AST, Platelet, Bilirubin, Albumin, INR, Sodium, Creatinine).

Model concordance index for all-cause mortality: 0.82, liver-related death: 0.84.

* P<0.05

** P<0.01

*** P<0.001

Liver-related death: Stage 3 (sHR:1.66, 95% CI:1.16–2.38, P = 0.006), Stage 4b (sHR:1.24, 95% CI:1.02–1.51, P = 0.034) and Stage 5 (sHR:1.85, 95% CI:1.58–2.18, P<0.001) had increased subdistribution hazards for liver-related death compared to Stage 1. There were no differences in the subdistribution hazard for liver-related mortality between patients in Stage 1 and Stage 2 (sHR:1.05, 95% CI:0.88–1.25, P = 0.628), or Stage 4a (sHR:1.15, 95% CI:0.83–1.59, P = 0.406). HCC was associated with an increased subdistribution hazard of liver-related death (sHR:2.85, 95% CI:2.49–3.27, P<0.001) (*Table 2*).

## Discussion

Until our current study, the Baveno VII 5-stage model of cirrhosis had not been described among the general cirrhosis population in the US. Although our model only accounts for 1-year outcomes, the observed mortality rate was more than a 4-fold increase compared to D’Amico et al.’s observations over 5-years [7]. These differences may be due to unique characteristics of each study’s cohort. D’Amico et al.’s prospective cohort study involved patients recruited between 1981–1984 from a single center in Italy who had lower comorbidity burdens and received very vigilant follow-up that included periodic assessments through scheduled endoscopy, abdominal ultrasonography, and laboratory testing [7]. In comparison, our comprehensive, contemporary cohort captures patients from a large metropolitan area that more closely represents how patients’ interface with the US healthcare system. Unfortunately, adherence to clinical practice guidelines in the US may be as low as 20% and can vary widely between institutions and providers [22, 23]. Quality improvement initiatives to increase institutional guideline-adherent care will improve cirrhosis outcomes; especially among those with compensated cirrhosis or with low MELD-Na scores [23–25]. These patients are often perceived to have lower disease burdens with more optimistic prognoses, though it has been found that nearly 50% of all patients with low MELD-Na cirrhosis still die from liver-related causes [13, 14, 26].

In a longitudinal and diverse US cohort, we found that the mortality among patients with cirrhosis in a US metropolitan area were much higher than those of prior reports [4, 7]. The 1-year mortality rate for patients in Stage 4 was more than two-fold higher compared to patients in Stage 3, rising from 7.3% to 16.3%. When additional decompensation complications occur (Stage 5), the 1-year mortality increased to 43.8%. Especially for those with variceal bleeding, ascites, and ≥2 decompensation complications, patients were more likely to die than they were to receive LT. Understanding the progression of cirrhosis through discrete stages suggest that interventions, notably LT referral and waitlisting, should be offered earlier in the disease process.

Classifying cirrhosis into discrete stages is essential for personalized care and more accurate risk stratification. Patients with ascites, variceal bleeding, and ≥2 different decompensating complications had 44.9%, 83.2%, and 95.2% increased risks of all-cause mortality, respectively compared to compensated patients without portal hypertension. Similarly, a study with a cohort of patients on the LT waitlist by Wedd et al. also found that the highest odds of mortality were among those with ≥2 decompensating complications (13-fold), variceal bleeding (8-fold), and non-bleeding complications (6-fold) compared to compensated patients without portal hypertension [9]. In our analyses, we observed that patients with HE had similar mortality risk compared to those with compensated cirrhosis. This is likely a limitation of HE captures in the electronic health records. Prospective studies offer a better qualification of clinically overt and subclinical HE. Nevertheless, the differential risks of clinical outcomes across cirrhosis stages may provide some insight on the optimal timing of interventions. Nevertheless, the differential risks of clinical outcomes across cirrhosis stages may provide some insight on the optimal timing of interventions. Future research, such as prospective study of cohorts (e.g., Liver Cirrhosis Network (LCN)) are needed to reduce the risks of disease progression.

Lastly, we found that patients were more likely to die than they were to receive a LT, even among those with ≥2 different decompensating complications, ascites, and variceal bleeding. Our data support previous recommendations that interventions that mitigate progression of the disease are likely to reduce mortality [7]. This includes consideration for liver transplantation (LT), because patients with more advanced stages face higher mortality as demonstrated with our data, as well as higher risk of denial or delisting due to increasing frailty [27] and systemic infections [28], as had been shown by others. Patients suffering from infectious complications are 5.2-fold more likely to be delisted than those without such complications [29]. We also recognize that LT is a limited resource that may not be available to all patients with cirrhosis due to a myriad of factors, including lack of access to specialized care and low organ availability. Our findings support efforts to expand the donor pool for LT through living donor organ donation [30, 31], use of machine perfusion for organs donated after circulatory death, and xenografts [32, 33]. Even for patients with MELD-Na scores of 11, living donor LT has been shown to have a 34% decrease in mortality compared to those on the waitlist [30]. This may provide a paradigm shift from prior notions that the survival benefit of LT was seen only among those with MELD scores of ≥15 and instead advocate that LT should be seriously considered earlier in the disease process [34]. Although further investigation is required, our findings may elucidate the optimal timing for LT referral and emphasize the importance of expanding the donor pool.

Our results should be interpreted within the context of our limitations. Because our study is retrospective in nature, causality cannot be implied. Next, the EHR codes used to define diagnoses inherently relies on the accuracy of coding administrators from each institution. However, these codes have been validated by the literature to have adequate specificity and positive predictive value for each associated diagnosis [16, 35]. It is also possible that complications occurred that were not captured by the HealthLNK network or coded by a care provider and could lead to an underestimation of the disease stage. This means that patients who have not appropriately been diagnosed, classified and coded by a physician will appear as Stage 1, that is, a patient might have more advanced cirrhosis, but no tests were performed to identify portal hypertension (e.g., upper endoscopy). To ameliorate this, we left-censored our cohort to improve the assumption that the first appearance of a cirrhosis diagnosis code would be the true index of cirrhosis diagnosis. Fourth, the observation period takes place at a time prior to the introduction of direct acting antiviral therapy for HCV. However, this allows our cohort to be more directly compared to prior studies [4, 7] and provides insight into the natural history of disease in the US population.

Lastly, it is also important to consider that other stages not currently included in the Baveno VII consensus model may arise as the scientific community gains a better understanding of liver disease. For example, acute-on-chronic liver failure (ACLF), a state of increasing organ failure and short-term mortality, has been described to potentially occur in both compensated or decompensated states [36]. In addition, considering pre-cirrhosis states, such as the stages of liver fibrosis, may also yield nuanced risks of clinical outcomes [37]. Nevertheless, this study is the first to investigate the disease trajectory of a large, longitudinal, population cohort of patients with cirrhosis in a diverse, urban US population. Future work with prospective longitudinal cohorts will further characterize cirrhosis progression.

In conclusion, at every stage of disease, the mortality among patients with cirrhosis in a metropolitan US population was much higher compared to prior reports. Patients with compensated cirrhosis without portal hypertension have 1-year mortality rates of 7.3%, and these rates increased more than 2-fold with the onset of one decompensation complication. Among patients with ≥2 different decompensation complications, the 1-year mortality rises to 43.8%. Furthermore, compared to compensated patients without portal hypertension, patients with ascites, variceal bleeding, and ≥2 different decompensating complications had 44.9%, 83.2%, and 95.2% increased risks of all-cause mortality, respectively. The Cirrhosis Progression Stages are vital in advancing our understanding of cirrhosis and may help guide timely interventions and patient management to prevent disease progression and mitigate mortality risk.

## Supporting information

S1 Table Inclusion ICD and CPT codes for patients with cirrhosis. (DOCX)

S2 Table Demographics of patients with cirrhosis by stage for those who died directly from each stage. (DOCX)

## Abbreviations

ANOVA: analysis of variance
CI: confidence interval
HBV: hepatitis B virus
HCC: Hepatocellular carcinoma
HCV: hepatitis C virus
HE: hepatic encephalopathy
HPS: hepatopulmonary syndrome
HRS: hepatorenal syndrome
ICD: International Classification of Diseases
LT: liver transplantation
MELD-: Na: Model for End-stage Liver Disease with Sodium
MASH: metabolic dysfunction associated steatohepatitis
NASH: non-alcoholic steatohepatitis
RMST: Restricted Mean Survival Time
SBP: spontaneous bacterial peritonitis
sHR: subdistribution hazard ratio
STROBE: Strengthening the Reporting of Observational Studies in Epidemiology
UNOS: United Network for Organ Sharing
US: United states
