# Supplementary material for: Disease trajectory and competing risks of patients with cirrhosis in the US
Source: PLoS One. 2025 Feb 14;20(2):e0313152. doi: 10.1371/journal.pone.0313152 (PMC11828360; doi:10.1371/journal.pone.0313152)
Supplement: S1 Table — (DOCX) [file pone.0313152.s001.docx]

**SUPPLEMENTARY MATERIALS:**

**Supplement Table 1: Inclusion ICD and CPT Codes for patients with cirrhosis.**

| **Criteria** | **Diagnosis/Procedure/Medication Code** | **Code Type** | **Code** |
| --- | --- | --- | --- |
| Cirrhosis | Diagnosis Code | ICD-9 | 571.2 |
|  |  | ICD-9 | 571.5 |
|  |  | ICD-9 | 571.6 |
| DECOMPENSATION EVENTS |  |  |  |
| Hepatic encephalopathy | Diagnosis Code | ICD-9 | 572.2 |
|  |  | ICD-9 | 070.0 |
|  |  | ICD-9 | 070.2 |
|  |  | ICD-9 | 070.22 |
|  |  | ICD-9 | 070.23 |
|  |  | ICD-9 | 070.4 |
|  |  | ICD-9 | 070.41 |
|  |  | ICD-9 | 070.44 |
|  |  | ICD-9 | 070.49 |
|  |  | ICD-9 | 070.6 |
|  |  | ICD-9 | 348.30 |
|  |  | ICD-9 | 348.31 |
|  |  | ICD-9 | 348.39 |
|  | Lactulose | Medication-RxCUI | 6218 |
|  |  | Medication-RxCUI | 216201 |
|  |  | Medication-RxCUI | 216928 |
|  |  | Medication-RxCUI | 217314 |
|  |  | Medication-RxCUI | 217928 |
|  |  | Medication-RxCUI | 1540455 |
|  |  | Medication-RxCUI | 6218 |
|  |  | Medication-RxCUI | 1251192 |
|  |  | Medication-RxCUI | 1251196 |
|  |  | Medication-RxCUI | 544452 |
|  |  | Medication-RxCUI | 544455 |
|  |  | Medication-RxCUI | 755470 |
|  |  | Medication-RxCUI | 1868572 |
|  |  | Medication-RxCUI | 1868578 |
|  |  | Medication-RxCUI | 544450 |
|  |  | Medication-RxCUI | 544453 |
|  |  | Medication-RxCUI | 567837 |
|  |  | Medication-RxCUI | 1868573 |
|  |  | Medication-RxCUI | 544451 |
|  |  | Medication-RxCUI | 544454 |
|  |  | Medication-RxCUI | 756967 |
|  |  | Medication-RxCUI | 1167052 |
|  |  | Medication-RxCUI | 1167053 |
|  |  | Medication-RxCUI | 1168758 |
|  |  | Medication-RxCUI | 1168759 |
|  |  | Medication-RxCUI | 1169806 |
|  |  | Medication-RxCUI | 1169807 |
|  |  | Medication-RxCUI | 1174250 |
|  |  | Medication-RxCUI | 1868574 |
|  |  | Medication-RxCUI | 104148 |
|  |  | Medication-RxCUI | 1251190 |
|  |  | Medication-RxCUI | 1251194 |
|  |  | Medication-RxCUI | 391937 |
|  |  | Medication-RxCUI | 1868568 |
|  |  | Medication-RxCUI | 1868576 |
|  |  | Medication-RxCUI | 336731 |
|  |  | Medication-RxCUI | 360554 |
|  |  | Medication-RxCUI | 1868570 |
|  |  | Medication-RxCUI | 378092 |
|  |  | Medication-RxCUI | 1162200 |
|  |  | Medication-RxCUI | 1162201 |
|  |  | Medication-RxCUI | 1868569 |
|  | Lactitol | Medication-RxCUI | 28395 |
|  |  | Medication-RxCUI | 2284606 |
|  |  | Medication-RxCUI | 1540455 |
|  |  | Medication-RxCUI | 28395 |
|  |  | Medication-RxCUI | 1305520 |
|  |  | Medication-RxCUI | 2284611 |
|  |  | Medication-RxCUI | 2284607 |
|  |  | Medication-RxCUI | 2284608 |
|  |  | Medication-RxCUI | 2284609 |
|  |  | Medication-RxCUI | 2284610 |
|  |  | Medication-RxCUI | 2284605 |
|  |  | Medication-RxCUI | 2284602 |
|  |  | Medication-RxCUI | 2284604 |
|  |  | Medication-RxCUI | 1162531 |
|  |  | Medication-RxCUI | 2284603 |
|  | Rifaximin | Medication-RxCUI | 35619 |
|  |  | Medication-RxCUI | 539786 |
|  |  | Medication-RxCUI | 539789 |
|  |  | Medication-RxCUI | 856666 |
|  |  | Medication-RxCUI | 539787 |
|  |  | Medication-RxCUI | 856665 |
|  |  | Medication-RxCUI | 539788 |
|  |  | Medication-RxCUI | 1186266 |
|  |  | Medication-RxCUI | 1186267 |
|  |  | Medication-RxCUI | 429662 |
|  |  | Medication-RxCUI | 856664 |
|  |  | Medication-RxCUI | 451308 |
|  |  | Medication-RxCUI | 856663 |
|  |  | Medication-RxCUI | 451309 |
|  |  | Medication-RxCUI | 1157434 |
|  |  | Medication-RxCUI | 1157435 |
| Variceal Bleeding | Diagnosis Code | ICD-9 | 456.0 |
|  |  | ICD-9 | 456.20 |
|  |  | ICD-9 | 578 |
|  |  | ICD-9 | 578.1 |
|  |  | ICD-9 | 578.9 |
|  | Procedure Code | CPT | 43243 |
|  |  | CPT | 43244 |
|  |  | CPT | 43255 |
| Ascites | Diagnosis Code | ICD-9 | 789.59 |
|  |  | ICD-9 | 511.89 |
|  |  | ICD-9 | 789.5 |
|  |  |  |  |
|  | Eplerenone | Medication-RxCUI | 298869 |
|  |  | Medication-RxCUI | 342280 |
|  |  | Medication-RxCUI | 298869 |
|  |  | Medication-RxCUI | 402105 |
|  |  | Medication-RxCUI | 402106 |
|  |  | Medication-RxCUI | 576107 |
|  |  | Medication-RxCUI | 576108 |
|  |  | Medication-RxCUI | 402363 |
|  |  | Medication-RxCUI | 1173417 |
|  |  | Medication-RxCUI | 1173418 |
|  |  | Medication-RxCUI | 351256 |
|  |  | Medication-RxCUI | 351257 |
|  |  | Medication-RxCUI | 351258 |
|  |  | Medication-RxCUI | 353386 |
|  |  | Medication-RxCUI | 353387 |
|  |  | Medication-RxCUI | 353388 |
|  |  | Medication-RxCUI | 378726 |
|  |  | Medication-RxCUI | 1161909 |
|  |  | Medication-RxCUI | 1161910 |
|  | Spironolactone | Medication-RxCUI | 9997 |
|  |  | Medication-RxCUI | 151317 |
|  |  | Medication-RxCUI | 17276 |
|  |  | Medication-RxCUI | 1943340 |
|  |  | Medication-RxCUI | 1008908 |
|  |  | Medication-RxCUI | 1008922 |
|  |  | Medication-RxCUI | 151194 |
|  |  | Medication-RxCUI | 2566848 |
|  |  | Medication-RxCUI | 324042 |
|  |  | Medication-RxCUI | 392462 |
|  |  | Medication-RxCUI | 814684 |
|  |  | Medication-RxCUI | 1943345 |
|  |  | Medication-RxCUI | 200817 |
|  |  | Medication-RxCUI | 200820 |
|  |  | Medication-RxCUI | 200825 |
|  |  | Medication-RxCUI | 208112 |
|  |  | Medication-RxCUI | 208116 |
|  |  | Medication-RxCUI | 2566860 |
|  |  | Medication-RxCUI | 2566864 |
|  |  | Medication-RxCUI | 2566868 |
|  |  | Medication-RxCUI | 1943341 |
|  |  | Medication-RxCUI | 2566855 |
|  |  | Medication-RxCUI | 2566863 |
|  |  | Medication-RxCUI | 2566867 |
|  |  | Medication-RxCUI | 565465 |
|  |  | Medication-RxCUI | 565468 |
|  |  | Medication-RxCUI | 565473 |
|  |  | Medication-RxCUI | 568840 |
|  |  | Medication-RxCUI | 568844 |
|  |  | Medication-RxCUI | 1943342 |
|  |  | Medication-RxCUI | 2566859 |
|  |  | Medication-RxCUI | 368071 |
|  |  | Medication-RxCUI | 369279 |
|  |  | Medication-RxCUI | 1174846 |
|  |  | Medication-RxCUI | 1174847 |
|  |  | Medication-RxCUI | 1174848 |
|  |  | Medication-RxCUI | 1174849 |
|  |  | Medication-RxCUI | 1943343 |
|  |  | Medication-RxCUI | 1943344 |
|  |  | Medication-RxCUI | 104230 |
|  |  | Medication-RxCUI | 104231 |
|  |  | Medication-RxCUI | 104232 |
|  |  | Medication-RxCUI | 104233 |
|  |  | Medication-RxCUI | 198222 |
|  |  | Medication-RxCUI | 198223 |
|  |  | Medication-RxCUI | 198224 |
|  |  | Medication-RxCUI | 198225 |
|  |  | Medication-RxCUI | 246399 |
|  |  | Medication-RxCUI | 250376 |
|  |  | Medication-RxCUI | 250377 |
|  |  | Medication-RxCUI | 313096 |
|  |  | Medication-RxCUI | 316727 |
|  |  | Medication-RxCUI | 316728 |
|  |  | Medication-RxCUI | 317508 |
|  |  | Medication-RxCUI | 360518 |
|  |  | Medication-RxCUI | 360519 |
|  |  | Medication-RxCUI | 360520 |
|  |  | Medication-RxCUI | 360521 |
|  |  | Medication-RxCUI | 370639 |
|  |  | Medication-RxCUI | 373944 |
|  |  | Medication-RxCUI | 373946 |
|  |  | Medication-RxCUI | 373948 |
|  |  | Medication-RxCUI | 379251 |
|  |  | Medication-RxCUI | 1161121 |
|  |  | Medication-RxCUI | 1161122 |
|  |  | Medication-RxCUI | 1161123 |
|  |  | Medication-RxCUI | 1161124 |
|  |  | Medication-RxCUI | 1162146 |
|  |  | Medication-RxCUI | 1162147 |
|  |  | Medication-RxCUI | 1162708 |
|  |  | Medication-RxCUI | 1162709 |
|  |  | Medication-RxCUI | 1164019 |
|  |  | Medication-RxCUI | 1164020 |
|  | Amiloride | Medication-RxCUI | 644 |
|  |  | Medication-RxCUI | 6958 |
|  |  | Medication-RxCUI | 644 |
|  |  | Medication-RxCUI | 1007938 |
|  |  | Medication-RxCUI | 1008209 |
|  |  | Medication-RxCUI | 1009094 |
|  |  | Medication-RxCUI | 1009137 |
|  |  | Medication-RxCUI | 153154 |
|  |  | Medication-RxCUI | 214212 |
|  |  | Medication-RxCUI | 608649 |
|  |  | Medication-RxCUI | 645371 |
|  |  | Medication-RxCUI | 812786 |
|  |  | Medication-RxCUI | 818351 |
|  |  | Medication-RxCUI | 819068 |
|  |  | Medication-RxCUI | 1298837 |
|  |  | Medication-RxCUI | 142424 |
|  |  | Medication-RxCUI | 977882 |
|  |  | Medication-RxCUI | 977881 |
|  |  | Medication-RxCUI | 368024 |
|  |  | Medication-RxCUI | 1178091 |
|  |  | Medication-RxCUI | 1178092 |
|  |  | Medication-RxCUI | 977880 |
|  |  | Medication-RxCUI | 977883 |
|  |  | Medication-RxCUI | 977887 |
|  |  | Medication-RxCUI | 977890 |
|  |  | Medication-RxCUI | 977907 |
|  |  | Medication-RxCUI | 977920 |
|  |  | Medication-RxCUI | 977945 |
|  |  | Medication-RxCUI | 977949 |
|  |  | Medication-RxCUI | 977950 |
|  |  | Medication-RxCUI | 977951 |
|  |  | Medication-RxCUI | 977959 |
|  |  | Medication-RxCUI | 977879 |
|  |  | Medication-RxCUI | 977886 |
|  |  | Medication-RxCUI | 977889 |
|  |  | Medication-RxCUI | 977891 |
|  |  | Medication-RxCUI | 370564 |
|  |  | Medication-RxCUI | 370638 |
|  |  | Medication-RxCUI | 370847 |
|  |  | Medication-RxCUI | 370849 |
|  |  | Medication-RxCUI | 370850 |
|  |  | Medication-RxCUI | 370851 |
|  |  | Medication-RxCUI | 379256 |
|  |  | Medication-RxCUI | 379257 |
|  |  | Medication-RxCUI | 1151590 |
|  |  | Medication-RxCUI | 1151591 |
|  |  | Medication-RxCUI | 1151594 |
|  |  | Medication-RxCUI | 1151595 |
|  |  | Medication-RxCUI | 1151596 |
|  |  | Medication-RxCUI | 1151597 |
|  |  | Medication-RxCUI | 1151600 |
|  |  | Medication-RxCUI | 1151601 |
|  |  | Medication-RxCUI | 1151604 |
|  |  | Medication-RxCUI | 1151605 |
|  |  | Medication-RxCUI | 1151607 |
|  |  | Medication-RxCUI | 1151608 |
|  |  | Medication-RxCUI | 1151613 |
|  |  | Medication-RxCUI | 1151614 |
|  |  | Medication-RxCUI | 1151615 |
|  | Furosemide | Medication-RxCUI | 4603 |
|  |  | Medication-RxCUI | 202991 |
|  |  | Medication-RxCUI | 2621024 |
|  |  | Medication-RxCUI | 1008347 |
|  |  | Medication-RxCUI | 1009094 |
|  |  | Medication-RxCUI | 392462 |
|  |  | Medication-RxCUI | 392464 |
|  |  | Medication-RxCUI | 392570 |
|  |  | Medication-RxCUI | 608054 |
|  |  | Medication-RxCUI | 818351 |
|  |  | Medication-RxCUI | 200801 |
|  |  | Medication-RxCUI | 200809 |
|  |  | Medication-RxCUI | 205732 |
|  |  | Medication-RxCUI | 2621028 |
|  |  | Medication-RxCUI | 2621025 |
|  |  | Medication-RxCUI | 565450 |
|  |  | Medication-RxCUI | 565458 |
|  |  | Medication-RxCUI | 566621 |
|  |  | Medication-RxCUI | 2621027 |
|  |  | Medication-RxCUI | 368487 |
|  |  | Medication-RxCUI | 1175647 |
|  |  | Medication-RxCUI | 1175648 |
|  |  | Medication-RxCUI | 104220 |
|  |  | Medication-RxCUI | 197731 |
|  |  | Medication-RxCUI | 197732 |
|  |  | Medication-RxCUI | 199610 |
|  |  | Medication-RxCUI | 246399 |
|  |  | Medication-RxCUI | 250708 |
|  |  | Medication-RxCUI | 251308 |
|  |  | Medication-RxCUI | 2621022 |
|  |  | Medication-RxCUI | 310429 |
|  |  | Medication-RxCUI | 313988 |
|  |  | Medication-RxCUI | 727574 |
|  |  | Medication-RxCUI | 727575 |
|  |  | Medication-RxCUI | 977890 |
|  |  | Medication-RxCUI | 977945 |
|  |  | Medication-RxCUI | 977959 |
|  |  | Medication-RxCUI | 315970 |
|  |  | Medication-RxCUI | 315971 |
|  |  | Medication-RxCUI | 315972 |
|  |  | Medication-RxCUI | 317377 |
|  |  | Medication-RxCUI | 331965 |
|  |  | Medication-RxCUI | 336694 |
|  |  | Medication-RxCUI | 360516 |
|  |  | Medication-RxCUI | 2621021 |
|  |  | Medication-RxCUI | 370847 |
|  |  | Medication-RxCUI | 373944 |
|  |  | Medication-RxCUI | 374196 |
|  |  | Medication-RxCUI | 727573 |
|  |  | Medication-RxCUI | 1151600 |
|  |  | Medication-RxCUI | 1151601 |
|  |  | Medication-RxCUI | 1162708 |
|  |  | Medication-RxCUI | 1162709 |
|  |  | Medication-RxCUI | 1162710 |
|  |  | Medication-RxCUI | 1162711 |
|  | Torsemide | Medication-RxCUI | 38413 |
|  |  | Medication-RxCUI | 71974 |
|  |  | Medication-RxCUI | 208076 |
|  |  | Medication-RxCUI | 208081 |
|  |  | Medication-RxCUI | 2589877 |
|  |  | Medication-RxCUI | 2589883 |
|  |  | Medication-RxCUI | 2589887 |
|  |  | Medication-RxCUI | 2589873 |
|  |  | Medication-RxCUI | 2589882 |
|  |  | Medication-RxCUI | 2589886 |
|  |  | Medication-RxCUI | 568804 |
|  |  | Medication-RxCUI | 568809 |
|  |  | Medication-RxCUI | 2589876 |
|  |  | Medication-RxCUI | 369285 |
|  |  | Medication-RxCUI | 1172416 |
|  |  | Medication-RxCUI | 1172417 |
|  |  | Medication-RxCUI | 2589874 |
|  |  | Medication-RxCUI | 2589875 |
|  |  | Medication-RxCUI | 198369 |
|  |  | Medication-RxCUI | 198370 |
|  |  | Medication-RxCUI | 198371 |
|  |  | Medication-RxCUI | 198372 |
|  |  | Medication-RxCUI | 250044 |
|  |  | Medication-RxCUI | 2589881 |
|  |  | Medication-RxCUI | 2589885 |
|  |  | Medication-RxCUI | 2589880 |
|  |  | Medication-RxCUI | 2589884 |
|  |  | Medication-RxCUI | 316842 |
|  |  | Medication-RxCUI | 316843 |
|  |  | Medication-RxCUI | 316844 |
|  |  | Medication-RxCUI | 316845 |
|  |  | Medication-RxCUI | 317521 |
|  |  | Medication-RxCUI | 335760 |
|  |  | Medication-RxCUI | 374168 |
|  |  | Medication-RxCUI | 1162752 |
|  |  | Medication-RxCUI | 1162753 |
|  | Bumetanide | Medication-RxCUI | 1808 |
|  |  | Medication-RxCUI | 203538 |
|  |  | Medication-RxCUI | 392534 |
|  |  | Medication-RxCUI | 645371 |
|  |  | Medication-RxCUI | 816562 |
|  |  | Medication-RxCUI | 205488 |
|  |  | Medication-RxCUI | 205489 |
|  |  | Medication-RxCUI | 205490 |
|  |  | Medication-RxCUI | 566394 |
|  |  | Medication-RxCUI | 566395 |
|  |  | Medication-RxCUI | 566396 |
|  |  | Medication-RxCUI | 369375 |
|  |  | Medication-RxCUI | 1168680 |
|  |  | Medication-RxCUI | 1168681 |
|  |  | Medication-RxCUI | 104222 |
|  |  | Medication-RxCUI | 197417 |
|  |  | Medication-RxCUI | 197418 |
|  |  | Medication-RxCUI | 197419 |
|  |  | Medication-RxCUI | 250660 |
|  |  | Medication-RxCUI | 431610 |
|  |  | Medication-RxCUI | 977951 |
|  |  | Medication-RxCUI | 315499 |
|  |  | Medication-RxCUI | 315500 |
|  |  | Medication-RxCUI | 315501 |
|  |  | Medication-RxCUI | 315502 |
|  |  | Medication-RxCUI | 332695 |
|  |  | Medication-RxCUI | 332696 |
|  |  | Medication-RxCUI | 360517 |
|  |  | Medication-RxCUI | 370850 |
|  |  | Medication-RxCUI | 371157 |
|  |  | Medication-RxCUI | 371158 |
|  |  | Medication-RxCUI | 438106 |
|  |  | Medication-RxCUI | 1151343 |
|  |  | Medication-RxCUI | 1151344 |
|  |  | Medication-RxCUI | 1151348 |
|  |  | Medication-RxCUI | 1151349 |
|  |  | Medication-RxCUI | 1151350 |
|  |  | Medication-RxCUI | 1151594 |
|  |  | Medication-RxCUI | 1151595 |
|  | Procedure Code | CPT | 49082 |
|  |  | CPT | 49083 |
| SBP | Diagnosis Code | ICD-9 | 567.0 |
|  |  | ICD-9 | 567.1 |
|  |  | ICD-9 | 567.21 |
|  |  | ICD-9 | 567.22 |
|  |  | ICD-9 | 567.23 |
|  |  | ICD-9 | 567.29 |
|  |  | ICD-9 | 567.31 |
|  |  | ICD-9 | 567.38 |
|  |  | ICD-9 | 567.39 |
|  |  | ICD-9 | 567.81 |
|  |  | ICD-9 | 567.82 |
|  |  | ICD-9 | 567.89 |
|  |  | ICD-9 | 567.9 |
| HRS | Diagnosis Code | ICD-9 | 572.4 |
| HPS | Diagnosis Code | ICD-9 | 573.5 |
| OUTCOMES OF INTEREST |  |  |  |
| HCC | Diagnosis Code | ICD-9 | 155.0 |
|  | Procedure Code | CPT | 79445 |
| Portal Hypertension | Diagnosis Code | ICD-9 | 572.3 |
| Varices without bleeding | Diagnosis Code | ICD-9 | 456.1 |
|  |  | ICD-9 | 456.21 |
|  | Procedure Code | CPT | 43235 |
|  |  | CPT | 43237 |
| TIPS | Procedure Code | CPT | 37182 |
|  |  | CPT | 37183 |
| ETIOLOGY/COMORBIDITIES |  |  |  |
| Hepatitis B | Diagnosis Code | ICD-9 | 070.20 |
|  |  | ICD-9 | 070.21 |
|  |  | ICD-9 | 070.22 |
|  |  | ICD-9 | 070.23 |
|  |  | ICD-9 | 070.30 |
|  |  | ICD-9 | 070.31 |
|  |  | ICD-9 | 070.32 |
|  |  | ICD-9 | 070.33 |
| Hepatitis C | Diagnosis Code | ICD-9 | 070.41 |
|  |  | ICD-9 | 070.44 |
|  |  | ICD-9 | 070.51 |
|  |  | ICD-9 | 070.54 |
|  |  | ICD-9 | 070.70 |
|  |  | ICD-9 | 070.71 |
| Alcohol | Diagnosis Code | ICD-9 | 571.1 |
|  |  | ICD-9 | 571.2 |
|  |  | ICD-9 | 571.3 |
|  |  | ICD-9 | 291.0 |
|  |  | ICD-9 | 291.1 |
|  |  | ICD-9 | 291.2 |
|  |  | ICD-9 | 291.3 |
|  |  | ICD-9 | 291.4 |
|  |  | ICD-9 | 291.5 |
|  |  | ICD-9 | 291.8 |
|  |  | ICD-9 | 291.81 |
|  |  | ICD-9 | 291.82 |
|  |  | ICD-9 | 291.89 |
|  |  | ICD-9 | 291.9 |
|  |  | ICD-9 | 303.00 |
|  |  | ICD-9 | 303.01 |
|  |  | ICD-9 | 303.02 |
|  |  | ICD-9 | 303.03 |
|  |  | ICD-9 | 303.9 |
|  |  | ICD-9 | 303.91 |
|  |  | ICD-9 | 303.92 |
|  |  | ICD-9 | 303.93 |
| Primary Biliary Cholangitis/Primary Sclerosing Cholangitis | Diagnosis Code | ICD-9 | 571.6 |
| Autoimmune | Diagnosis Code | ICD-9 | 571.42 |
| Cardiac | Diagnosis Code | ICD-9 | 573.0 |
| Genetic | Diagnosis Code | ICD-9 | 273.4 |
|  |  | ICD-9 | 275.01 |
|  |  | ICD-9 | 275.1 |
|  |  | ICD-9 | 275.03 |
| Obesity | Diagnosis Code | ICD-9 | 278.00 |
|  |  | ICD-9 | 278.01 |
|  |  | ICD-9 | 278.02 |
|  |  | ICD-9 | 278.03 |
| Diabetes, and/or diabetic complications | Diagnosis Code | ICD-9 | 250.00 |
|  |  | ICD-9 | 250.01 |
|  |  | ICD-9 | 250.02 |
|  |  | ICD-9 | 250.03 |
|  |  |  |  |
|  |  | ICD-9 | 250.10 |
|  |  | ICD-9 | 250.11 |
|  |  | ICD-9 | 250.12 |
|  |  | ICD-9 | 250.13 |
|  |  | ICD-9 | 250.20 |
|  |  | ICD-9 | 250.21 |
|  |  | ICD-9 | 250.22 |
|  |  | ICD-9 | 250.23 |
|  |  | ICD-9 | 250.30 |
|  |  | ICD-9 | 250.31 |
|  |  | ICD-9 | 250.32 |
|  |  | ICD-9 | 250.33 |
|  |  | ICD-9 | 250.80 |
|  |  | ICD-9 | 250.81 |
|  |  | ICD-9 | 250.82 |
|  |  | ICD-9 | 250.83 |
|  |  | ICD-9 | 250.90 |
|  |  | ICD-9 | 250.91 |
|  |  | ICD-9 | 250.92 |
|  |  | ICD-9 | 250.93 |
|  |  | ICD-9 | 648.01 |
|  |  | ICD-9 | 775.2 |
|  |  | ICD-9 | 250.40 |
|  |  | ICD-9 | 250.41 |
|  |  | ICD-9 | 250.42 |
|  |  | ICD-9 | 250.43 |
|  |  | ICD-9 | 250.50 |
|  |  | ICD-9 | 250.51 |
|  |  | ICD-9 | 250.52 |
|  |  | ICD-9 | 250.53 |
|  |  | ICD-9 | 250.60 |
|  |  | ICD-9 | 250.61 |
|  |  | ICD-9 | 250.62 |
|  |  | ICD-9 | 250.63 |
|  |  | ICD-9 | 250.70 |
|  |  | ICD-9 | 250.71 |
|  |  | ICD-9 | 250.72 |
|  |  | ICD-9 | 250.73 |
| Hypertension and/or hypertensive complications | Diagnosis Code | ICD-9 | 401.0 |
|  |  | ICD-9 | 401.1 |
|  |  | ICD-9 | 401.9 |
|  |  | ICD-9 | 642.0 |
|  |  | ICD-9 | 402.00 |
|  |  | ICD-9 | 402.01 |
|  |  | ICD-9 | 402.10 |
|  |  | ICD-9 | 402.11 |
|  |  | ICD-9 | 402.90 |
|  |  | ICD-9 | 402.91 |
|  |  | ICD-9 | 403.00 |
|  |  | ICD-9 | 403.01 |
|  |  | ICD-9 | 403.10 |
|  |  | ICD-9 | 403.11 |
|  |  | ICD-9 | 403.90 |
|  |  | ICD-9 | 403.91 |
|  |  | ICD-9 | 404.00 |
|  |  | ICD-9 | 404.01 |
|  |  | ICD-9 | 404.02 |
|  |  | ICD-9 | 404.03 |
|  |  | ICD-9 | 404.10 |
|  |  | ICD-9 | 404.11 |
|  |  | ICD-9 | 404.12 |
|  |  | ICD-9 | 404.13 |
|  |  | ICD-9 | 404.90 |
|  |  | ICD-9 | 404.91 |
|  |  | ICD-9 | 404.92 |
|  |  | ICD-9 | 404.93 |
|  |  | ICD-9 | 405.01 |
|  |  | ICD-9 | 405.09 |
|  |  | ICD-9 | 405.11 |
|  |  | ICD-9 | 405.19 |
|  |  | ICD-9 | 405.91 |
|  |  | ICD-9 | 405.99 |
|  |  | ICD-9 | 642.10 |
|  |  | ICD-9 | 642.11 |
|  |  | ICD-9 | 642.12 |
|  |  | ICD-9 | 642.13 |
|  |  | ICD-9 | 642.14 |
|  |  | ICD-9 | 642.20 |
|  |  | ICD-9 | 642.21 |
|  |  | ICD-9 | 642.22 |
|  |  | ICD-9 | 642.23 |
|  |  | ICD-9 | 642.24 |
|  |  | ICD-9 | 642.70 |
|  |  | ICD-9 | 642.71 |
|  |  | ICD-9 | 642.72 |
|  |  | ICD-9 | 642.73 |
|  |  | ICD-9 | 642.74 |
|  |  | ICD-9 | 642.90 |
|  |  | ICD-9 | 642.91 |
|  |  | ICD-9 | 642.92 |
|  |  | ICD-9 | 642.93 |
|  |  | ICD-9 | 642.94 |
| Dyslipidemia | Diagnosis Code | ICD-9 | 272.0 |
|  |  | ICD-9 | 272.1 |
|  |  | ICD-9 | 272.2 |
|  |  | ICD-9 | 272.3 |
|  |  | ICD-9 | 272.4 |
|  |  | ICD-9 | 272.5 |
|  |  | ICD-9 | 272.6 |
|  |  | ICD-9 | 272.7 |
|  |  | ICD-9 | 272.8 |
|  |  | ICD-9 | 272.9 |
