# Supplementary material for: Disease trajectory and competing risks of patients with cirrhosis in the US
Source: PLoS One. 2025 Feb 14;20(2):e0313152. doi: 10.1371/journal.pone.0313152 (PMC11828360; doi:10.1371/journal.pone.0313152)
Supplement: S2 Table — (DOCX) [file pone.0313152.s002.docx]

**SUPPLEMENTARY MATERIALS:**

**Supplemental Table 2. Demographics of patients with cirrhosis by stage for those who died directly from each stage.**

|  | **Compensated Cirrhosis** | |  | **Decompensated Cirrhosis** | | |  |
| --- | --- | --- | --- | --- | --- | --- | --- |
| **Characteristic** | **Death Directly from Stage I   (n = 442 )** | **Death Directly from Stage II  (n = 134 )** |  | **Death Directly from Stage III   (n = 74)** | **Death Directly from Stage IV  (n = 548 )** | **Death Directly from Stage V   (n = 1,490 )** | **p-value** |
| Age, year, mean (±SD) | 61.74 (11.27) | 58.72 (9.88) |  | 58.91 (12.71) | 61.27 (12.11) | 58.38 (11.26) | <0.001 |
| Female, n (%) | 150 (33.94%) | 38 (28.36%) |  | 26 (35.14%) | 203 (37.04%) | 553 (37.11%) | 0.2624 |
| Race, n (%) |  |  |  |  |  |  |  |
| Non-Hispanic White | 187 (42.31%) | 57 (42.54%) |  | 25 (33.78%) | 278 (50.73%) | 716 (48.05%) | 0.0060 |
| Non-Hispanic Black | 109 (24.66%) | 30 (22.39%) |  | 31 (41.89%) | 119 (21.72%) | 345 (23.15%) | 0.0045 |
| Hispanic | 20 (4.52%) | 9 (6.72%) |  | 3 (4.05%) | 26 (4.74%) | 78 (5.23%) | 0.2699 |
| Asian | 8 (1.81%) | 3 (2.24%) |  | 0 (0%) | 8 (1.46%) | 40 (2.68%) | 0.8436 |
| Other | 118 (26.7%) | 35 (26.12%) |  | 15 (20.27%) | 117 (21.35%) | 311 (20.87%) | 0.0755 |
| Insurance, n (%) |  |  |  |  |  |  |  |
| Medicare/Medicaid | 242 (54.75%) | 58 (43.28%) |  | 38 (51.35%) | 312 (56.93%) | 780 (52.35%) | 0.0595 |
| Private | 98 (22.17%) | 27 (20.15%) |  | 10 (13.51%) | 116 (21.17%) | 416 (27.92%) | 0.0020 |
| Other | 102 (23.08%) | 49 (36.57%) |  | 26 (35.14%) | 120 (21.9%) | 294 (19.73%) | <0.001 |
| Follow-up time, mean (±SD) | 42.51 (13.83) | 44.18 (19.07) |  | 43.78 (19.44) | 40.54 (13.3) | 38.58 (12.6) | 0.0288 |
| Charlson Comorbidity Index, mean (±SD) | 5.56 (3.66) | 8.82 (3.08) |  | 7.55 (3.49) | 7.34 (3.81) | 8.56 (2.94) | <0.001 |
| Etiology, n (%) |  |  |  |  |  |  |  |
| Hepatitis B | 33 (7.47%) | 16 (11.94%) |  | 5 (6.76%) | 33 (6.02%) | 132 (8.86%) | 0.2504 |
| Hepatitis C | 142 (32.13%) | 49 (36.57%) |  | 26 (35.14%) | 177 (32.3%) | 516 (34.63%) | 0.3548 |
| Alcohol-related | 146 (33.03%) | 58 (43.28%) |  | 37 (50%) | 203 (37.04%) | 785 (52.68%) | <0.001 |
| MASH | 93 (21.04%) | 21 (15.67%) |  | 14 (18.92%) | 133 (24.27%) | 270 (18.12%) | 0.0010 |
| Cholestasis | 7 (1.58%) | 1 (0.75%) |  | 3 (4.05%) | 12 (2.19%) | 34 (2.28%) | 0.6257 |
| Autoimmune | 2 (0.45%) | 1 (0.75%) |  | 1 (1.35%) | 7 (1.28%) | 24 (1.61%) | 0.6207 |
| Genetic | 1 (0.23%) | 1 (0.75%) |  | 0 (0%) | 6 (1.09%) | 23 (1.54%) | 0.2649 |
| Labs |  |  |  |  |  |  |  |
| MELD-Na, median (IQR) | 15 (11.5,20) | 15.5 (12.25,21.75) |  | 18 (13,22) | 17 (13,22) | 22 (16,29) | <0.001 |
| FIB-4 score, median (IQR) | 3.42 (1.8,5.09) | 8.58 (5.24,13.1) |  | 6.15 (2.61,10.28) | 4.00 (2.4,6.93) | 6.73 (3.79,10.8) | <0.001 |
| Fib-4>2.67, n (%) | 80 (18.1%) | 89 (66.42%) |  | 29 (39.19%) | 175 (31.93%) | 682 (45.77%) | <0.001 |
| Platelets, median (IQR) | 190 (143.25,255.75) | 86 (61,107) |  | 116 (67,227.5) | 168 (118.75,234) | 116 (76,181) | <0.001 |
| Platelets<100, n (%) | 0 (0%) | 73 (54.48%) |  | 17 (22.97%) | 39 (7.12%) | 333 (22.35%) | <0.001 |
| AST, median (IQR) | 55 (34,105) | 74 (44,113) |  | 61 (36,140) | 61 (36,107.25) | 78 (47.5,129.5) | <0.001 |
| ALT, median (IQR) | 35 (21,61) | 39 (25,59) |  | 30 (20,63) | 34 (20,60.25) | 38 (23,65) | 0.1106 |
| Albumin, median (IQR) | 2.9 (2.3,3.33) | 2.8 (2.4,3.4) |  | 2.6 (2.1,3.1) | 2.7 (2.1,3.1) | 2.4 (2,2.9) | <0.001 |
| HCC, n (%) | 93 (21.04%) | 31 (23.13%) |  | 13 (17.57%) | 143 (26.09%) | 306 (20.54%) | <0.001 |
| LT, n (%) | 2 (0.45%) | 0 (0%) |  | 0 (0%) | 0 (0%) | 4 (0.27%) | 0.5967 |
| Death, n (%) | 442 (100%) | 134 (100%) |  | 74 (100%) | 548 (100%) | 1490 (100%) |  |
| Liver-related | 227 (51.36%) | 78 (58.21%) |  | 33 (44.59%) | 309 (56.39%) | 1091 (73.22%) | <0.001 |
| Non-Liver | 163 (36.88%) | 50 (37.31%) |  | 26 (35.14%) | 166 (30.29%) | 263 (17.65%) | <0.001 |
| Non-descript | 52 (11.76%) | 6 (4.48%) |  | 15 (20.27%) | 73 (13.32%) | 136 (9.13%) | <0.001 |

ALT: Alanine transaminase, AST: aspartate transaminase, FIB-4= fibrosis-4 score, HCC: hepatocellular carcinoma, LT: liver transplant, MASH: metabolic dysfunction associated steatohepatitis, SBP: spontaneous bacterial peritonitis, Stage 1: compensated cirrhosis without portal hypertension, Stage 2: compensated cirrhosis with portal hypertension, Stage 3: variceal bleeding, Stage 4: non-bleeding decompensation complication, Stage 5: ≥2 different decompensation complications.
